# Cytokine-induced changes in the gene expression profile of a human cerebral microvascular endothelial cell-line, hCMEC/D3

**DOI:** 10.1186/2045-8118-10-27

**Published:** 2013-09-19

**Authors:** Miguel Alejandro Lopez-Ramirez, David Kingsley Male, Chunfang Wang, Basil Sharrack, Dongsheng Wu, Ignacio Andres Romero

**Affiliations:** 1 Department of Life, Health & Chemical Sciences, The Open University, Milton Keynes MK7 6AA, UK; 2 Department of Neurology, Sheffield Teaching Hospitals NHS Trust, University of Sheffield, Sheffield, UK; 3 Present address; Yale Cardiovascular Research Center, Section of Cardiovascular Medicine, Yale University, School of Medicine, New Haven, USA

**Keywords:** Blood–brain barrier, Transcriptome, Cytokines, Transporter systems, Inter-brain endothelial junctions, Integrin-focal adhesions

## Abstract

**Background:**

The human cerebral microvascular endothelial cell line, hCMEC/D3, has been used extensively to model the blood–brain barrier (BBB) *in vitro*. Recently, we reported that cytokine-treatment induced loss of brain endothelial barrier properties. In this study, we further determined the gene expression pattern of hCMEC/D3 cells in response to activation with TNFα and IFNγ.

**Findings:**

Using a microarray approach, we observed that expression of genes involved in the control of barrier permeability, including inter-brain endothelial junctions (e.g. claudin-5, MARVELD-2), integrin-focal adhesions complexes (e.g. integrin β1, ELMO-1) and transporter systems (e.g. ABCB1, SLC2A1), are altered by pro-inflammatory cytokines.

**Conclusions:**

Our study shows that previously-described cytokine-induced changes in the pattern of gene expression of endothelium are reproduced in hCMEC/D3 cells, suggesting that this model is suitable to study inflammation at the BBB, while at the same time it has provided insights into novel key molecular processes that are altered in brain endothelium during neuroinflammation, such as modulation of cell-to-matrix contacts.

## Findings

### Introduction

Cerebral endothelial cells (CEC) constitute the major element of the highly specialized vasculature of the central nervous system (CNS) that contributes to the blood–brain barrier (BBB). *In vitro* models have complemented animal and human studies in our current understanding of BBB function. To date, the best characterized *in vitro* human BBB model is based on the human cerebral microvascular endothelial cell line, hCMEC/D3. Indeed, recent studies on hCMEC/D3 cells have elucidated bacterial and viral CNS invasion mechanisms [1,2], leukocyte trafficking [3], regulation of transporters [4,5] and signalling pathways controlling CEC paracellular permeability [6-9]. Here, we provide further insights into the molecular mechanisms associated with barrier dysfunction by analyzing the gene expression pattern in cytokine-activated hCMEC/D3 cells using mRNA microarray analysis.

## Materials and methods

### Culture conditions

hCMEC/D3 cells were cultured in EGM-2 MV medium (Lonza, Slough Wokingham, UK) and supplemented with the following components obtained from the manufacturer: 0.025% (v/v) rhEGF, 0.025% (v/v) VEGF, 0.025% (v/v) IGF, 0.1% (v/v) rhFGF, 0.1% (v/v) gentamycin, 0.1% (v/v) ascorbic acid, 0.04% (v/v) hydrocortisone and 2.5% (v/v) foetal bovine serum (FBS). Cells were seeded onto flasks supplied by Greiner Bio-one (Gloucestershire, UK), previously coated with collagen, and maintained at 37°C in 95% air and 5% CO2 until confluence.

### RNA extraction and mRNA microarray analysis

hCMEC/D3 cells were grown on collagen-coated six-well plates and treated with 10 ng/ml of TNFα and IFNγ (R&D systems, Abingdon, Oxon, UK) for 24 h, while control cells received the vehicle solution. Cells were washed once with pre-warmed Hank’s balanced salts solution. Total RNA from three biological replicates was isolated using miRNeasy mini kit (Qiagen, Crawley, West Sussex, UK) according to the manufacturer’s protocols. The RNA was re-suspended using RNase-free water. The quantity (NanoDrop 1000 spectrophotometer) and the quality (2100 Bioanalyzer, RNA 6000 Pico LabChip; Agilent, Palo Alto, CA, USA) were assessed for each RNA sample. For each biological sample 100 ng of total RNA was used, obtained from approximately 1.5 ×10^6^ cells. For mRNA profiling, Ambion® TotalPrep 96 RNA Amplification kit and Illumina hybridisation protocols were carried out by Cambridge Genomic Services (Cambridge, UK). The analysis was run using LUMI and LIMMA packages (R Bioconductor). A quantile normalization was performed and a number of quality plots were generated to assess the quality of the data. Differences between control and treated cells were estimated when a gene signal in two or more replicates is at the background level. Instead of using numerical values, we indicated these genes as upregulated (+) or downregulated (-) in cytokine-treated cells. Detailed procedures and complete data are available at the Gene Expression Omnibus (http://www.ncbi.nlm.nih.gov/geo) under accession number GSE45880.

### Quantitative RT-PCR

Complementary DNA was obtained using reverse transcriptase (Promega, Madison, WI) with random primers according to the manufacturer’s protocol. SYBR Green real-time PCR (Qiagen, Manchester, UK) was used to determine the relative levels of the genes analyzed (Table 1). The reaction was then placed in a thermal cycler (DNA engine Opticon 2; Bio-Rad, Hercules, CA, USA) using an initial step at 95°C for 15 min, followed by 40 cycles (15 s at 94°C, 30 s at 55°C, and 30 s at 72°C). The 2 ^–ΔΔCT^ method was used for analysis of the data.

**Table 1:** Primers used for quantitative RT-PCR on hCMEC/D3 cells

**Category**	**Gene**	**Forward and reverse primers**	**Gene entrez**	**Start**	**Stop**
Cell-cell contact	CDH5	**F:** 5′CAGATCTCCGCAATAGACAAGG3′	NM_001795.2	1563	1585
		**R:** 5′CGTGATTATCCGTGAGGGTAAAG3′		1637	1660
	MARVELD2	**F**: 5′GTACTCGTGGTTGCTGGATTAG3′	NM_001038603.2	840	862
		**R**: 5′GCCACCAATTAGAGTCCAGAAG3′		921	943
	ANXA2	**F**: 5′GAAACAGCCATCAAGACCAAAG3′	NM_001002858.2	254	276
		**R**: 5′TGGTAGGCGAAGGCAATATC3′		335	355
Cell-to-matrix adhesion	ITGB1	**F**: 5′CATGTTGTGGAGAATCCAGAGT3′	NM_002211.3	2367	2389
		**R**: 5′GCAGTAATGCAAGGCCAATAAG3′		2445	2467
	ELMO1	**F**: 5′GACCTGGGTTGAGATGATATGG3′	NM_014800.10	4407	4429
		**R**: 5′GAGTGCTCTGATGGGAAGAAG3′		4483	4504
	FHL2	**F**: 5′CAGAAACTCACTGGTGGACAA3′	NM_201555.1	792	813
		**R**: 5′ATTCCTGGCACTTGGATGAG3′		870	890
Chemokines/cell adhesion molecules	CCL2	**F**: 5′GGCTGAGACTAACCCAGAAAC3′	NM_002982.3	13	34
		**R**: 5′GAATGAAGGTGGCTGCTATGA3′		108	129
	VCAM-1	**F**: 5′CATTTGACAGGCTGGAGATAGA3′	M73255.1	1217	1239
		**R**: 5′CTCTTGGTTTCCAGGGACTT3′		1297	1317
	CEACAM1	**F**: 5′CTACCTGTAGGATCAGGGTCTAA3′	NM_001712.4	1991	2014
		**R**: 5′CTAGTTGCTTCTAGTGGGTTCTC3′		2069	2092
Transporters	ABCB1	**F**: 5′TGCAGGTACCATACAGAAACTC3′	NM_000927.4	3264	3286
		**R**: 5′ACCGGAAACATCCAGCATAG3′		3349	3369
	SLC2A1	**F**: 5′GGACAGGCTCAAAGAGGTTATG3′	NM_006516.2	3217	3239
		**R**: 5′AGGAGGTGGGTGGAGTTAAT 3′		3309	3329
	SLC2A3	**F**: 5′CTTAGTTCTCACTGTTCCCTCTG3′	NM_006931.2	3397	3420
		**R**: 5′TCCCAAAGTGCTGGGATTAC3′		3480	3500
Internal standard	ACTB	**F:** 5′GGACCTGACTGACTACCTCAT 3′	NM_001101.3	633	654
		**R:** 5′CGTAGCACAGCTTCTCCTTAAT 3′		718	740

### Bioinformatic analysis

DAVID bioinformatics [10] tool was used to create the functional annotation clustering from microarray analysis by using KEGG pathways. Transcripts with more than one probe represented in the mRNA microarray that showed contradictory results in the direction of changes in expression levels between control cells and cells treated with TNFα and IFNγ, were considered as not showing any changes.

### Statistical analysis

The statistical analysis for the mRNA microarray was performed using pairwise comparisons and false discovery rate. The data set was filtered before normalisation, removing any probes that were not successfully detected (detection *P* was less than 0.01) in at least one sample. Statistical significance was considered if *P* was less than 0.01 for KEGG analysis or 0.05 for barrier-related gene analysis determined by LIMMA software. For quantitative RT-PCR, statistical significance was considered if *P* was less than 0.05 as determined by paired, one-tailed Student’s *t* test.

## Results

### Pro-inflammatory cytokine modulation of gene expression profile of cultured human brain endothelium

**Figure 1:**
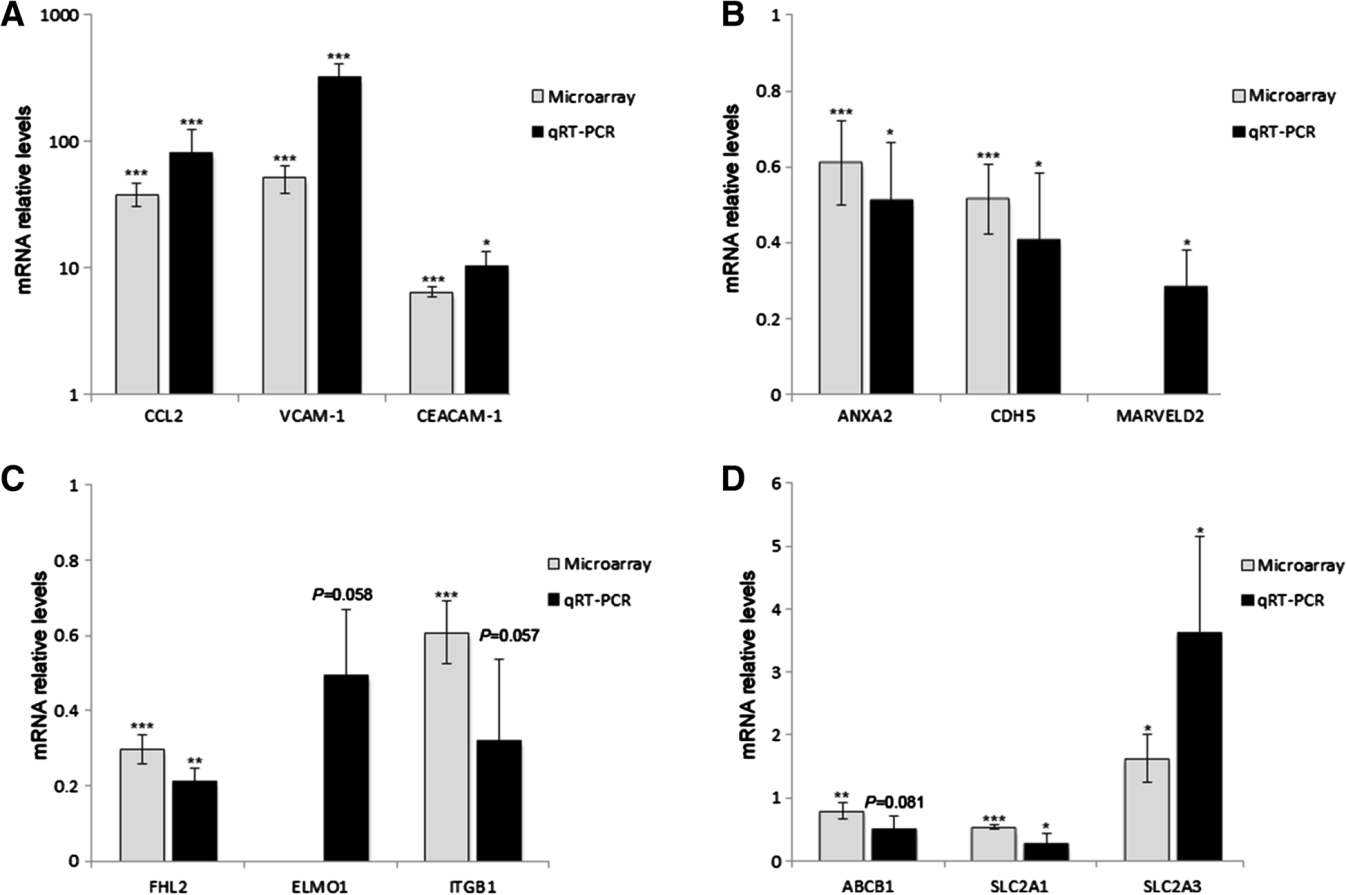
**Validation of mRNA microarray findings by quantitative RT-PCR.** Levels of 12 genes selected for validation were determined by quantitative RT-PCR (qRT-PCR). qRT-PCR data parallel that from the mRNA microarray analysis for genes associated with **(A)** chemokines/CAMs, **(B)** cell-cell contacts, **(C)** cell-matrix adhesion, and **(D)** transporters. Control values were normalized to one and results are expressed as levels in cytokine-treated cells relative to those in unstimulated cells. Actin-β was used as an internal standard. *P < 0.05, **P < 0.01, ***P < 0.001 compared to unstimulated cells. Note that for ELMO and MARVELD2 the signal intensity in cytokine-treated cells was detected at the background level in the mRNA microarray analysis.

In order to identify the major molecular processes involved in cytokine-induced altered CEC phenotype, we analyzed the changes in gene expression in hCMEC/D3 cells after stimulation with TNFα and IFNγ for 24 h using mRNA array. By considering the transcripts that were positively regulated with at least a two-fold change over unstimulated cells (404 genes, *P* < 0.01) we identified three statistically significant biological process associated with antigen presentation (human leukocyte antigen, HLA)/cellular adhesion (cell adhesion molecules, CAM) (20 genes), cytokine/chemokine activity (28 genes) and cytokine-induced signalling pathways (16 genes) (Additional file 1: Table S1 TNFα and IFNγ-induced modulation of gene expression profile of cultured human brain endothelium). Other minor functional categories identified were associated with apoptosis [11] and complement/coagulation cascades [12] (Additional file 1: Table S1). In addition, Additional file 1: Table S1, shows cytokine-induced increase of 24 mRNA transcripts associated with interleukin and decrease of 7 genes in hCMEC/D3 cells. We validated cytokine-induced changes in the levels of 3 mRNAs in hCMEC/D3 cells including CCL2, VCAM-1 and CEACAM-1 (Figure 1A). Overall, these results indicate that proinflammatory cytokines trigger changes in the gene expression pattern of hCMEC/D3 cells with roles in facilitating leukocyte adhesion and transmigration across the brain endothelium.

### Pro-inflammatory cytokine modulation of transcripts related to BBB permeability

Recently, we reported that TNFα alone or in combination with IFNγ trigger signalling pathways that result in loss of barrier properties by negative modulation of cell-cell contacts [8]. Hence, we investigated genes involved in the control of the paracellular pathway of brain endothelium. Additional file 2: Table S2 (TNFα and IFNγ-induced modulation of transcripts related to BBB permeability) shows cytokine-induced decrease of 27 mRNA transcripts associated with the junctional complex molecules (JCM), and an increase of 14 genes in hCMEC/D3 cells. Furthermore, brain endothelial barrier properties may be determined by cellular attachment to the extracellular matrix [13]. We observed that stimulation with TNFα and IFNγ induced changes in the expression pattern of focal adhesion (FA) genes in hCMEC/D3 cells. Cytokines reduced the expression of 45 transcripts and increased of 43 transcripts out of the 126 FA genes identified in hCMEC/D3 cells (Additional file 2: Table S2). In addition, 10 integrin genes out of the 18 integrin transcripts identified in hCMEC/D3 cells were downregulated by cytokines, whereas only integrin-α_v_ was increased. The qRT-PCR analysis confirms the downregulation of 6 genes associated with cell-cell contacts and cell-matrix adhesion (Figure 1B, 1C).

Another category that defines the functionality of BBB is the expression of transporters. We observed that TNFα and IFNγ reduced the expression of 50 transporters at the mRNA level, including ABCB1 (P-glycoprotein, P-gp), ABCG2 and SLC2A1 (Glut-1) (Figure 1D, Additional file 2: Table S2). Conversely, 25 mRNA transcripts that code for transporters were increased, including the chemoresistant tranporter ABCB8 and the glucose transporter SLC2A3 (Glut-3) (Figure 1D, Additional file 2: Table S2). These results indicate that TNFα and IFNγ remarkably affect the brain endothelial gene expression pattern of cell-cell contacts, cell-matrix adhesion and transport barrier systems.

## Discussion

Here we showed an over-view of the cellular process altered in cytokine-activated brain endothelium using mRNA array analysis. Previous studies have shown that microvascular and macrovascular human endothelium have several differences in response to inflammatory stimulus however some similarities are also shared [12,14]. In our analysis, we observed that some of the early changes in the gene expression pattern induced by TNFα in HUVECs [11,12,14] were also seen during long-term exposure to TNFα and IFNγ in hCMEC/D3 cells. These include the positive regulation of chemokines, CAMs, apoptosis genes, complement and coagulation cascades [11,12,14]. Furthermore, among the CAMs upregulated by TNFα and IFNγ we identified CEACAM-1, a molecule involved in angiogenesis and vascular permeability [15], although its role in brain endothelial barrier function have not been explored yet. In addition, we recently reported that the ability of CEC to respond to inflammatory stimuli by changing the pattern of gene expression is in part controlled by transcriptional activity [8,9] and in part at the post-transcriptional level via small non coding RNAs termed microRNAs [16,17]. For instance, miR-155 overexpression in hCMEC/D3 cells reflects the activated state of CEC induced by TNFα and IFNγ, these include upregulation of genes associated with antigen presentation, CAMs, complement pathways, cytokine activity and barrier breakdown [17].

One molecular mechanism associated with cytokine-induced endothelial barrier dysfunction is reorganization of both cytoplasmic and transmembrane JCM from the cell-cell contacts [8,18]. Here, we observed by gene expression profiling in hCMEC/D3 cells that stimulation with TNFα and IFNγ for 24 h altered mRNA levels of several JCM including claudin-5 and MARVELD-2 (also known as tricellulin), both previously reported to be enriched in CECs [19]. Indeed, we recently reported that changes in paracellular permeability and transendothelial electrical resistance correlated with changes in the expression of claudin-5 at the transcript and protein level [8,9]. Another molecular mechanism associated with loss of endothelial barrier integrity is rearrangement of integrin-focal adhesion complexes [20,21]. Our data supports the hypothesis that FA constitute a novel pathway that is critical for BBB maintenance [13] as many FA components were modulated by cytokines in CECs. For instance, integrin ß1 serves as a platform to establish focal contacts and is downregulated by cytokines in hCMEC/D3 cells. In addition, loss of integrin ß1 might also affect claudin-5 protein levels [22] and thus the brain endothelial barrier [8].

We have considered some potential criticisms of the interpretations given above. First, high concentrations of TNFα and IFNγ induce an increase in CEC paracellular permeability associated with caspase-3/-7 activation and apoptotic cell death [8]. We cannot discard the possibility that some of the effects observed in this analysis might be secondary to a small loss of cell viability. However, our gene expression profile and the altered expression of BBB-associated genes in hCMEC/D3 cells after cytokine treatment correlate well with a recent report [23]. A second possible drawback is that most of the changes in the gene expression pattern reported here need to be further validated. However, the altered levels observed in 12 genes after cytokine-treatment within 4 functional pathways were validated by qRT-PCR. We observed that three genes analyzed, ELMO1, ITGB1 and ABCB1, were always downregulated after cytokine-treatment, but their fold changes were variable. In addition, some of the functional consequences of TNFα and IFNγ on CEC have been reported. For instance, in agreement with our findings, Poller et al. [4] showed that TNFα reduced expression of BCRP (ABCG2) mRNA levels, protein levels and functional activity [4]. Additionally, we previously demonstrated that CCL2, CXCL10 and CCL5 protein expression is upregulated by stimulation with TNFα and IFNγ in hCMEC/D3 cells [24] and that these chemokines have also been detected in MS plaques [24]. Similar to our results Pan et al. [25] reported that TNFα induced an increased in IL-15 and its receptor, IL15RA, in rat brain endothelial cells suggesting an important role of this interleukin in the brain endothelial response to cytokines [25] that is conserved between species.

In summary, pro-inflammatory cytokines might alter the highly selective barrier permeability of brain endothelial cells by establishing a new pattern of gene expression. Changes in expression of CEC genes involve biological processes associated with regulation of leukocyte infiltration, inter-brain endothelial junctions, integrin-focal adhesions and transport systems. This analysis provides insight into key molecular and cellular processes altered during neuroinflammation.

## Abbreviations

BBB: Blood–brain barrier; hCMEC/D3: The human cerebral microvascular endothelial cell line; CEC: Cerebral endothelial cells; CNS: Central nervous system; CAM: Cell adhesion molecules; HLA: Human leukocyte antigen; JCM: Junctional complex molecules; FA: Focal adhesion.

## Competing interests

The authors declare that they have no competing interests.

## Authors’ contributions

MALR, performed research, analyzed and interpreted the data; IAR, DKM, DW, BS, and CW, provided support with analysis of data and interpretation of results; DW and CW performed validation of array analysis; MALR, DKM, and IAR designed the study and wrote the manuscript. All authors read and approved the final manuscript.

## Supplementary Material

Additional file 1 TNFα and IFNγ-induced modulation of gene expression profile of cultured human brain endothelium. Click here for file

Additional file 2 TNFα and IFNγ-induced modulation of transcripts related to BBB permeability. Click here for file
