# Supplementary material for: Cytokine-induced changes in the gene expression profile of a human cerebral microvascular endothelial cell-line, hCMEC/D3
Source: Fluids Barriers CNS. 2013 Sep 19;10:27. doi: 10.1186/2045-8118-10-27 (PMC3849656; doi:10.1186/2045-8118-10-27)
Supplement: Additional file 1 — TNFα and IFNγ-induced modulation of gene expression profile of cultured human brain endothelium. [file 2045-8118-10-27-S1.pdf]

Table S1. TNFα and IFNγ-induced modulation of gene expression profile of cultured human brain endothelium

CATEGORY	TERM	COUNT	PValue	FOLD ENR. BONFERRONI	BENJAMINI
KEGG_PATHWAY	HLA/CAM	20	1.91E-08	2.31E-06	5.79E-07
KEGG_PATHWAY	Cytokine/Chemokine activity	28	2.80E-08	3.375373382	3.38E-06
KEGG_PATHWAY	cytokine-induced signalling pathways	16	4.34E-07	5.26E-05	4.78E-06
KEGG_PATHWAY	Complement and coagulation cascades	8	0.005613018	0.493934113	0.041674727
KEGG_PATHWAY	Apoptosis	9	0.005713793	0.500102261	0.039964864

continue Table S1

Chemokines			
**ACCESSION**	**SYMBOL**	**FOLD-CHANGE OR REGULATION**	**ADJ.P.VAL**
NM_001040138.1	CKLF	-1.560	0.000035
NM_001040139.1	CKLF	-1.445	0.000385
NM_000609.4	CXCL12	-	1.77E-05
NM_001338.3	CXADR	-	7.07E-05
NM_145898.1	CCL23	-	0.018426597
NM_199168.2	CXCL12	-	2.97E-06
NM_001565.2	CXCL10	167.240	3.42E-09
NM_002036.2	DARC	4.855	1.27E-05
NM_002089.3	CXCL2	3.576	1.64E-07
NM_002982.3	CCL2	38.559	2.00E-07
NM_002985.2	CCL5	35.166	2.84E-11
NM_002996.3	CX3CL1	25.551	3.90E-09
NM_022059.1	CXCL16	3.175	5.92E-08
NM_001511.1	CXCL1	+	4.98E-06
NM_002416.1	CXCL9	+	9.19E-12
NM_002993.2	CXCL6	+	5.27E-07
NM_002994.3	CXCL5	+	8.82E-05
NM_004591.1	CCL20	+	2.30E-08
NM_005623.2	CCL8	+	1.02E-11
**Interleukins**			
**ACCESSION**	**SYMBOL**	**FOLD-CHANGE OR REGULATION**	**ADJ.P.VAL**
NM_138284.1	IL17D	-2.571	1.15E-05
NM_001560.2	IL13RA1	-2.185	1.79E-06
NM_004512.3	IL11RA	-1.874	6.35E-05
NM_000572.2	IL10	-1.299	0.008634538
NM_004512.3	IL11RA	-1.223	0.013733677
NM_000565.2	IL6R	-1.200	0.025617916
NM_000565.2	IL6R	-1.129	0.074339811
NM_152456.1	IL34	1.224	0.03412512
NM_173843.1	IL1RN	1.251	0.016566058

NM_004843.2	IL27RA	1.350	0.002341774
NM_000882.2	IL12A	1.375	0.000232668
NM_153701.1	IL12RB1	1.391	0.000282523
NM_000585.2	IL15	1.399	0.001212931
NM_153701.1	IL12RB1	1.562	2.60E-05
NM_002185.2	IL7R	1.592	3.60E-05
NM_000575.3	IL1A	1.665	1.38E-05
NM_002183.2	IL3RA	1.703	8.68E-05
NM_002189.2	IL15RA	1.904	2.33E-05
NM_003855.2	IL18R1	2.087	0.000237793
NM_003856.2	IL1RL1	2.340	7.18E-05
NM_173042.2	IL18BP	2.583	4.27E-07
NM_000600.1	IL6	3.225	3.61E-07
NM_002189.2	IL15RA	3.575	1.91E-08
NM_003856.2	IL1RL1	3.928	1.36E-05
NM_172374.1	IL4I1	9.878	3.42E-09
NM_173042.2	IL18BP	19.573	7.35E-10
NM_001012636.1	IL32	23.343	1.05E-10
NM_001012633.1	IL32	24.945	5.18E-10
NM_000584.2	IL8	56.262	3.62E-09
**Cell adhesion molecules**			
**ACCESSION**	**SYMBOL**	**FOLD-CHANGE OR REGULATION**	**ADJ.P.VAL**
NM_000442.3	PECAM1	-1.706	0.021712033
NM_001099786.1	ICAM2	-1.537	0.002696545
NM_002162.2	ICAM3	-1.319	0.003817802
NM_032801.3	JAM3	-1.577	0.000226073
NM_138961.1	ESAM	-2.639	0.000525608
NM_001024912.1	CEACAM1	5.388	3.22E-08
NM_001024912.1	CEACAM1	6.520	5.56E-08
NM_001078.2	VCAM1	52.031	5.93E-09
NM_005010.3	NRCAM	1.961	3.19E-06
NM_005010.3	NRCAM	2.310	3.55E-06
NM_175573.1	*ADRM1	1.490	0.000372749
NM_000201.1	ICAM1	+	0.000178645
NM_001712.3	CEACAM1	+	9.98E-08
NM_001627.2	ALCAM	N	0.822056005
NM_006500.2	MCAM	N	0.121193353
NM_024003.1	L1CAM	N	0.111091182
NM_016174.3	CERCAM	N	0.603205212
NM_021189.2	CADM3	N	0.678687481
NM_175573.1	*ADRM1	N	0.097286675

- Indicates downregulation below detection levels in cytokine-treated cells, + indicates upregulation from below detection levels in control cells, N indicates no changes between control and cytokine-treated cells and * indicates transcript showing different results with different probes.
