# Supplementary material for: Cytokine-induced changes in the gene expression profile of a human cerebral microvascular endothelial cell-line, hCMEC/D3
Source: Fluids Barriers CNS. 2013 Sep 19;10:27. doi: 10.1186/2045-8118-10-27 (PMC3849656; doi:10.1186/2045-8118-10-27)
Supplement: Additional file 2 — TNFα and IFNγ-induced modulation of transcripts related to BBB permeability. [file 2045-8118-10-27-S2.pdf]

Table S2. TNFα and IFNγ-induced modulation of transcripts related to BBB permeability

Structural<br>tight junctions			
**ACCESSION**	**SYMBOL**	**FOLD-CHANGE OR REGULATION**	**ADJ.P.VAL**
NM_003277.2	CLDN5	-2.086	0.006518769
NM_032801.3	JAM3	-1.577	0.000226073
NM_014343.1	CLDN15	-1.530	7.59E-05
NM_152353.1	CLDND2	-	0.003523584
NM_144724.1	MARVELD2	-	0.01440481
NM_021101.3	CLDN1	3.190	1.13E-05
NM_012129.2	CLDN12	1.274	0.01151853
NM_194284.2	CLDN23	1.519	0.000742419
NM_001307.3	CLDN7	1.432	0.001070304
NM_144504.1	JAM1	1.337	0.011583927
NM_016946.3	JAM1	+	0.002479634
NM_012130.2	CLDN14	N	0.173320936
NM_001001346.2	CLDN20	N	0.772348864
NM_001040181.1	CLDND1	N	0.500496117
NM_001040181.1	CLDND1	N	0.663446269
**Structural<br>adherens junctions**			
**ACCESSION**	**SYMBOL**	**FOLD-CHANGE OR REGULATION**	**ADJ.P.VAL**
NM_001792.2	CDH2	-1.764	8.45E-05
NM_001795.2	CDH5	-1.944	0.000393004
NM_015480.1	PVRL3	-1.713	4.40E-05
NM_001042724.1	PVRL2	2.781	3.37E-07
NM_002856.2	PVRL2	2.577	1.02E-06
NM_001042724.1	PVRL2	2.443	2.10E-06
**Gap junctions**			
**ACCESSION**	**SYMBOL**	**FOLD-CHANGE OR REGULATION**	**ADJ.P.VAL**
NM_000165.3	GJA1	-1.710	0.00441142
NM_005266.4	GJA5	+	0.027873225
**Cytoplasmic<br>tight junctions**			
**ACCESSION**	**SYMBOL**	**FOLD-CHANGE OR REGULATION**	**ADJ.P.VAL**
NM_001002857.1	ANXA2	-1.634	1.31E-05
NM_001002858.1	ANXA2	-1.393	0.001044011
NM_001002857.1	ANXA2	-1.277	0.016785097
NM_018054.4	*ARHGAP17	-1.225	0.015350988
NM_000075.2	CDK4	-1.479	8.62E-05

NM_022474.2	MPP5	-1.235	0.005262576
NM_001037281.1	*PARD6A	-1.503	0.000216379
NM_000314.4	PTEN	-1.632	3.18E-05
NM_002867.2	RAB3B	-1.291	0.006151095
NM_080604.1	TJAP1	-1.367	0.005183602
NM_133646.2	ZAK	-2.784	2.23E-06
NM_016653.2	ZAK	-1.290	0.001124454
NM_018054.4	*ARHGAP17	1.396	0.001435095
NM_032866.3	CGNL1	1.404	0.002714655
NM_002870.2	RAB13	1.139	0.082771835
NM_004819.2	SYMPK	1.251	0.002643408
NM_175610.2	*TJP1	1.271	0.002446291
NM_016948.2	*PARD6A	N	0.137275818
NR_001562.1	ANXA2P1	N	0.84613659
NM_003257.3	*TJP1	N	0.21981928
NM_004817.2	TJP2	N	0.445415986
NM_201629.1	TJP2	N	0.232800829
NM_032387.3	WNK4	N	0.94206841
**Cytoplasmic adherens junctions**			
**ACCESSION**	**SYMBOL**	**FOLD-CHANGE OR REGULATION**	**ADJ.P.VAL**
NM_001903.2	CTNNA1	-1.393	0.00055142
NM_003798.1	CTNNAL1	-1.348	0.001586702
NM_020248.2	CTNNBIP1	-1.491	0.000365347
NM_002230.1	*JUP	-1.532	0.002773573
NM_015356.3	*SCRIB	-1.587	0.000439375
NM_030877.3	CTNNBL1	2.067	1.92E-06
NM_001098209.1	CTNNB1	N	0.075733212
NM_001085458.1	CTNND1	N	0.821672833
NM_021991.1	*JUP	N	0.059370165
NM_002230.1	*JUP	N	0.88181532
NM_001005242.1	PKP2	N	0.726001649
NM_015356.3	*SCRIB	N	0.073029717
**Desmosomal**			
**ACCESSION**	**SYMBOL**	**FOLD-CHANGE OR REGULATION**	**ADJ.P.VAL**
NM_003628.3	PKP4	-1.462	0.000734378
NM_001005242.1	PKP2	N	0.726001649
**Less known**			
**ACCESSION**	**SYMBOL**	**FOLD-CHANGE OR REGULATION**	**ADJ.P.VAL**
NM_002436.2	MPP1	-1.621	4.84E-05
NM_175573.1	*ADRM1	1.490	0.000372749
NM_001042486.1	DLGAP4	1.831	3.04E-05

NM_183006.2	DLGAP4	+	0.000535124
NM_175573.1	*ADRM1	N	0.097286675
NM_018979.2	WNK1	N	0.1944283
**Cell-cell and cell-matrix adhesion regulatory molecules**			
**ACCESSION**	**SYMBOL**	**FOLD-CHANGE OR REGULATION**	**ADJ.P.VAL**
NM_002473.3	MYH9	-1.262	0.003504242
NM_002737.2	PRKCA	-2.240	1.43E-05
NM_145040.2	PRKCDBP	-1.622	0.002914552
NM_005400.2	PRKCE	-1.328	0.005357462
NM_006255.3	PRKCH	-1.634	0.000626576
NM_001001329.1	*PRKCSH	-1.288	0.002282669
NM_006908.3	RAC1	-1.303	0.001496582
NM_006254.3	PRKCD	1.521	0.000400994
NM_001001329.1	*PRKCSH	1.239	0.006727286
NM_006097.3	MYL9	N	0.925567532
NM_002740.5	PRKCI	N	0.232800829
NM_006257.2	PRKCQ	N	0.237578572
**Focal adhesion molecules**			
**ACCESSION**	**SYMBOL**	**FOLD-CHANGE OR REGULATION**	**ADJ.P.VAL**
NM_000118.1	ENG	-1.550	0.003975044
NM_000314.4	PTEN	-1.632	3.18E-05
NM_001034954.1	SORBS1	-1.469	0.002387067
NM_001040023.1	SIRPA	-1.733	6.89E-05
NM_001567.2	INPPL1	-1.275	0.02243745
NM_002211.2	ITGB1	-1.615	9.43E-05
NM_002715.2	PPP2CA	-1.426	0.00058442
NM_002737.2	PRKCA	-2.240	1.43E-05
NM_002745.4	MAPK1	-1.304	0.005300108
NM_002834.3	PTPN11	-1.529	0.000280123
NM_002840.3	PTPRF	-2.545	1.71E-06
NM_002890.1	RASA1	-1.339	0.000520442
NM_003603.4	SORBS2	-1.941	0.0053419
NM_004840.2	ARHGEF6	-1.569	0.000148197
NM_004987.3	LIMS1	-1.238	0.007645093
NM_005022.2	PFN1	-1.258	0.003484453
NM_005157.3	ABL1	-1.379	0.000364235
NM_005188.2	CBL	-1.262	0.014968071
NM_005343.2	HRAS	-1.542	0.000381485
NM_005507.2	CFL1	-1.589	0.000327233
NM_005541.3	INPP5D	-1.278	0.002972738
NM_005578.2	LPP	-1.243	0.045530455
NM_005607.3	PTK2	-1.452	0.000402379
NM_005633.2	SOS1	-0.773	0.002931232

NM_005775.3	SORBS3	-1.462	0.000127173
NM_006098.4	GNB2L1	-1.201	0.007904323
NM_006148.1	LASP1	-1.435	0.000178301
NM_006908.3	RAC1	-1.303	0.001496582
NM_012383.3	OSTF1	-1.229	0.033866015
NM_012398.1	PIP5K1C	-1.252	0.013761439
NM_014000.2	VCL	-1.314	0.004007924
NM_014288.3	ITGB3BP	-1.223	0.008476532
NM_015927.3	*TGFB1I1	-1.172	0.02873836
NM_030768.2	ILKAP	-1.202	0.006085583
NM_031892.1	SH3KBP1	-1.438	0.000488666
NM_031892.1	SH3KBP1	-1.253	0.005463696
NM_032349.2	NUDT16L1	-1.281	0.003187908
NM_032876.4	JUB	-1.308	0.00348688
NM_033669.1	ITGB1	-1.487	8.47E-05
NM_130440.1	PTPRF	-1.731	0.000145252
NM_144641.1	PPM1M	-1.491	0.00035368
NM_201557.2	FHL2	-3.372	3.00E-06
NM_001024227.1	*ARF1	-1.417	0.001168351
NM_002205.2	ITGA5	-1.412	0.001895908
NM_201555.1	FHL2	-2.985	1.19E-05
NM_014800.9	ELMO1	-	0.006460642
NM_182966.2	NEDD9	-	0.034363415
NM_001005361.1	DNM2	1.578	7.68E-05
NM_001005376.1	PLAUR	1.426	0.002664706
NM_001005376.1	PLAUR	2.018	4.92E-06
NM_001007067.1	SDCBP	1.994	6.22E-05
NM_001009944.1	*PKD1	1.346	0.005494299
NM_001014794.1	ILK	1.648	0.000762502
NM_001024912.1	CEACAM1	5.388	3.22E-08
NM_001024912.1	CEACAM1	6.520	5.56E-08
NM_001030002.1	GRB7	1.367	0.000686216
NM_001101.2	ACTB	1.190	0.038649507
NM_001101.2	ACTB	1.345	0.003744627
NM_001102.2	ACTN1	1.823	2.09E-05
NM_001380.3	DOCK1	1.291	0.00404949
NM_002037.3	FYN	1.534	7.13E-05
NM_002350.1	LYN	2.762	2.93E-05
NM_002356.5	MARCKS	3.090	7.00E-06
NM_002613.3	PDPK1	1.221	0.02789832
NM_002659.2	PLAUR	1.873	7.67E-06
NM_002999.2	SDC4	2.088	6.69E-06
NM_003029.3	*SHC1	1.595	0.000179266
NM_003461.4	*ZYX	1.610	0.00017673
NM_004343.2	CALR	1.877	0.000347059
NM_004945.2	DNM2	1.265	0.01216074

NM_005231.2	*CTTN	1.412	0.000149676
NM_007118.2	TRIO	1.161	0.028280631
NM_012289.3	KEAP1	1.167	0.037837545
NM_012289.3	KEAP1	1.243	0.003418584
NM_013327.3	PARVB	1.292	0.009697523
NM_014030.3	GIT1	1.266	0.008369366
NM_080422.1	PTPN2	1.702	3.68E-05
NM_080840.2	PTPRA	1.669	3.87E-05
NM_080841.2	PTPRA	1.287	0.009354278
NM_133452.1	RAVER1	1.186	0.017037939
NM_138565.1	CTTN	1.230	0.011990068
NM_138957.2	*MAPK1	1.250	0.006030541
NM_153047.1	FYN	1.321	0.002273278
NM_153047.1	FYN	1.373	0.00126248
NM_153048.1	FYN	1.374	0.00169859
NM_198291.1	SRC	1.479	0.019884085
NM_198793.2	CD47	1.760	5.64E-05
NM_213662.1	STAT3	2.032	3.95E-06
NM_018212.4	ENAH	+	0.000108801
NM_002842.2	PTPRH	+	0.010259729
NM_016735.1	LIMK1	+	0.007277601
NM_080548.3	PTPN6	+	0.000861258
NM_198679.1	*RAPGEF1	+	0.023215649
NM_001712.3	CEACAM1	+	9.98E-08
NM_001777.3	CD47	+	0.002002172
NM_173176.1	PTK2B	+	0.000603398
NM_001009944.1	*PKD1	N	0.204714631
NM_001012751.1	ABI1	N	0.112867902
NM_001014431.1	AKT1	N	0.428090371
NM_001014432.1	AKT1	N	0.614732784
NM_001024215.1	FBLIM1	N	0.419750606
NM_001024228.1	*ARF1	N	0.844816268
NM_001040439.1	MAPK8IP3	N	0.602397655
NM_001042669.1	ARHGAP24	N	0.089744057
NM_001540.2	HSPB1	N	0.305812639
NM_001664.2	RHOA	N	0.163803357
NM_001753.3	CAV1	N	0.537110186
NM_002086.3	GRB2	N	0.111551053
NM_002086.3	GRB2	N	0.360850757
NM_002568.3	PABPC1	N	0.106303017
NM_002662.2	PLD1	N	0.472204154
NM_002827.2	PTPN1	N	0.896460364
NM_002835.2	PTPN12	N	0.252203962
NM_002859.1	PXN	N	0.506933977
NM_003029.3	*SHC1	N	0.709483874
NM_003047.2	SLC9A1	N	0.061360242

NM_003302.2	TRIP6	N	0.428897626
NM_003370.3	VASP	N	0.215638649
NM_003373.3	VCL	N	0.624753153
NM_003380.2	VIM	N	0.386599257
NM_003461.4	*ZYX	N	0.106684709
NM_003567.2	BCAR3	N	0.929493241
NM_003626.2	PPFIA1	N	0.881184849
NM_004078.1	CSRP1	N	0.850706868
NM_004103.3	PTK2B	N	0.333261543
NM_004383.1	CSK	N	0.630174165
NM_004517.2	ILK	N	0.102913089
NM_004811.1	LPXN	N	0.112462183
NM_004986.2	KTN1	N	0.122708036
NM_005186.2	CAPN1	N	0.063157228
NM_005231.2	*CTTN	N	0.074725558
NM_005406.2	ROCK1	N	0.40201846
NM_005544.1	IRS1	N	0.163857962
NM_005731.2	ARPC2	N	0.122708036
NM_006218.2	PIK3CA	N	0.264232251
NM_006285.2	TESK1	N	0.119267193
NM_006289.2	TLN1	N	0.288554931
NM_006403.2	NEDD9	N	0.182655248
NM_007313.2	ABL1	N	0.59260795
NM_014567.2	BCAR1	N	0.942230601
NM_015385.2	SORBS1	N	0.584573262
NM_015927.3	*TGFB1I1	N	0.060748342
NM_016081.3	PALLD	N	0.229530611
NM_016823.2	CRK	N	0.404508177
NM_016823.2	CRK	N	0.94902115
NM_018222.2	PARVA	N	0.073119041
NM_021979.2	HSPA2	N	0.557673767
NM_022648.3	TNS1	N	0.722009512
NM_031268.4	PDPK1	N	0.401427859
NM_031305.2	ARHGAP24	N	0.134836292
NM_031305.2	ARHGAP24	N	0.424925028
NM_080792.2	SIRPA	N	0.701909421
NM_138957.2	*MAPK1	N	0.559872758
NM_144573.3	NEXN	N	0.120713601
NM_177423.1	PPFIA1	N	0.467308111
NM_177423.1	PPFIA1	N	0.684812784
NM_182811.1	PLCG1	N	0.080232319
NM_182811.1	PLCG1	N	0.091308098
NM_182926.2	KTN1	N	0.08562298
NM_198679.1	*RAPGEF1	N	0.096612205
**Integrins**			

ACCESSION	SYMBOL	FOLD-CHANGE OR REGULATION	ADJ.P.VAL
NM_002203.3	ITGA2	-1.289	0.008224136
NM_002213.3	ITGB5	-3.933	7.97E-08
NM_002213.3	ITGB5	-2.939	2.31E-07
NM_003637.3	ITGA10	-1.477	0.004964577
NM_018463.2	ITFG2	-1.354	0.000506939
NM_022334.3	ITGB1BP1	-1.486	0.001005761
NM_033668.1	ITGB1	-1.648	2.98E-05
NM_000210.2	ITGA6	-	0.000848493
NM_001005619.1	ITGB4	-	3.19E-05
NM_181501.1	ITGA1	-	0.001461038
NM_002210.2	ITGAV	1.408	0.000348199
NM_000211.2	ITGB2	N	0.101053652
NM_000212.2	ITGB3	N	0.056217426
NM_002204.1	ITGA3	N	0.445418908
NM_002208.4	ITGAE	N	0.115840878
NM_004763.3	ITGB1BP1	N	0.814806184
NM_032039.1	ITFG3	N	0.103667795
NM_181469.1	ITGB4BP	N	0.178223145
**Solute carriers**			
**ACCESSION**	**SYMBOL**	**FOLD-CHANGE OR REGULATION**	**ADJ.P.VAL**
NM_003038.2	SLC1A4	-1.161	0.0261998
NM_020246.2	SLC12A9	-1.283	0.0026724
NM_004695.2	SLC16A5	-1.813	0.0000073
NM_005415.3	SLC20A1	-1.358	0.0004818
NM_006749.3	SLC20A2	-1.524	0.0008527
NM_007105.1	SLC22A18AS	-1.362	0.0026595
NM_144712.2	SLC23A3	-1.295	0.0055581
NM_012140.3	SLC25A10	-1.508	0.0000428
NM_003951.2	*SLC25A14	-1.236	0.0082199
NM_006358.2	SLC25A17	-1.216	0.0089357
NM_021734.3	SLC25A19	-1.561	0.0000706
NM_024103.2	SLC25A23	-2.086	0.0000026
NM_052901.2	*SLC25A25	-1.165	0.0223075
NM_001039355.1	*SLC25A29	-1.284	0.0060305
NM_018155.1	SLC25A36	-1.206	0.0382539
NM_016016.1	SLC25A39	-1.610	0.0000736
NM_001151.2	SLC25A4	-1.497	0.0000812
NM_178526.1	SLC25A42	-1.680	0.0000118
NM_001152.1	SLC25A5	-1.373	0.0003450
XM_001132495.1	SLC26A11	-1.456	0.0012279
NM_134426.2	SLC26A6	-1.242	0.0044100
NM_198580.1	SLC27A1	-1.317	0.0007227
NM_024330.1	SLC27A3	-1.777	0.0000167

NM_001078174.1	*SLC29A1	-1.251	0.0135777
NM_006516.1	SLC2A1	-1.860	0.0000760
NM_020062.3	SLC2A4RG	-1.517	0.0000689
NM_014580.3	SLC2A8	-1.916	0.0000031
NM_021194.2	SLC30A1	-1.292	0.0073565
NM_022902.2	SLC30A5	-1.631	0.0000224
NM_006345.3	SLC30A9	-1.543	0.0001645
NM_017515.3	SLC35F2	-1.325	0.0063314
NM_001467.4	SLC37A4	-1.627	0.0001123
NM_030674.3	SLC38A1	-1.626	0.0005662
NM_030674.3	SLC38A1	-1.280	0.0232198
NM_020342.1	SLC39A10	-1.595	0.0000179
NM_144564.4	*SLC39A3	-1.352	0.0007355
NM_001008485.1	SLC41A3	-1.831	0.0000040
NM_017836.3	SLC41A3	-1.588	0.0000348
NM_080546.3	*SLC44A1	-1.947	0.0000025
NM_020428.2	SLC44A2	-2.700	0.0000002
NM_033102.2	SLC45A3	-1.491	0.0001324
NM_181785.2	SLC46A3	-1.225	0.0478647
NM_016615.2	SLC6A13	-1.156	0.0403559
NM_182767.3	SLC6A15	-1.734	0.0000442
NM_018057.4	SLC6A15	-1.280	0.0227109
NM_182767.3	SLC6A15	-1.190	0.0185960
NM_003045.3	SLC7A1	-1.646	0.0002440
NM_003486.5	SLC7A5	-1.517	0.0053040
NM_004252.2	SLC9A3R1	-1.372	0.0002678
NM_005630.1	SLCO2A1	-2.326	0.0000242
NM_012254.1	SLC27A5	-	0.0277369
NM_145176.2	SLC2A12	-	0.0014351
NM_000617.1	*SLC11A2	1.335	0.0360691
NM_016582.1	*SLC15A3	3.003	0.0000011
NM_003705.2	SLC25A12	1.195	0.0126111
NM_031212.3	SLC25A28	2.100	0.0000100
NM_016612.2	SLC25A37	3.034	0.0000211
NM_017875.1	SLC25A38	1.142	0.0494433
NM_138773.1	SLC25A46	1.276	0.0398410
NM_006931.1	SLC2A3	1.631	0.0174551
NM_017585.2	SLC2A6	5.787	0.0000000
NM_133496.3	SLC30A7	1.307	0.0014251
NM_001860.2	SLC31A2	2.686	0.0000002
NM_004733.2	*SLC33A1	1.137	0.0376683
NM_001032289.1	*SLC35A2	1.180	0.0217454
NM_080670.2	SLC35A4	1.436	0.0002946
NM_173073.2	*SLC35C2	1.161	0.0332070
NM_007001.1	SLC35D2	1.166	0.0182932
NM_018964.3	SLC37A1	1.278	0.0076248

NM_032295.2	*SLC37A3	1.209	0.0276825
NM_001037984.1	*SLC38A10	1.139	0.0446279
NM_033518.1	SLC38A5	1.538	0.0000749
NM_014437.3	SLC39A1	1.299	0.0013815
NM_015359.2	SLC39A14	1.485	0.0007532
NM_012319.2	SLC39A6	1.247	0.0192512
NM_014096.2	SLC43A3	1.491	0.0004092
NM_017611.2	SLC43A3	1.855	0.0000221
NM_033051.2	SLC46A2	1.207	0.0115226
NM_003040.2	SLC4A2	1.347	0.0028388
NM_021095.1	SLC5A6	1.401	0.0001766
NM_001008539.2	SLC7A2	4.933	0.0000002
NM_003982.2	SLC7A7	1.379	0.0291232
NM_016354.3	SLCO4A1	3.570	0.0000001
NM_001032289.1	*SLC35A2	+	0.0302666
NM_032148.2	SLC41A2	+	0.0000148
NM_004785.3	SLC9A3R2	+	0.0000314
NM_015945.10	*SLC35C2	N	0.0720592
NM_019848.2	SLC10A3	N	0.5533214
NM_001029998.2	SLC10A7	N	0.1320659
NM_001046.2	*SLC12A2	N	0.1222428
NM_005072.3	SLC12A4	N	0.3090568
NM_022829.4	*SLC13A3	N	0.7075612
NM_145648.1	SLC15A4	N	0.2676937
NM_213606.1	SLC16A12	N	0.7482783
NM_004207.2	SLC16A3	N	0.6702074
NM_194255.1	SLC19A1	N	0.3001186
NM_005628.1	SLC1A5	N	0.2172643
NM_002555.3	SLC22A18	N	0.1248867
NM_003059.2	SLC22A4	N	0.2243845
NM_003060.2	SLC22A5	N	0.3506278
NM_005847.3	SLC23A1	N	0.4033362
NM_004727.2	SLC24A1	N	0.0729433
NM_024959.2	SLC24A6	N	0.3357290
NM_005984.1	SLC25A1	N	0.7569795
NM_003562.3	SLC25A11	N	0.2111496
NM_014251.1	SLC25A13	N	0.9144456
NM_022810.1	*SLC25A14	N	0.2957475
NM_024698.4	SLC25A22	N	0.2956596
NM_013386.3	SLC25A24	N	0.0948117
NM_052901.2	*SLC25A25	N	0.0563015
NM_173471.2	SLC25A26	N	0.8582356
NM_001009937.1	SLC25A26	N	0.2777276
NM_001039355.1	*SLC25A29	N	0.1560603
NM_002635.2	SLC25A3	N	0.0705441
NM_201520.1	SLC25A35	N	0.5303580

NM_018843.2	SLC25A40	N	0.3330651
NM_145305.1	SLC25A43	N	0.0732068
NM_014655.1	SLC25A44	N	0.8671586
NM_001078174.1	*SLC29A1	N	0.2136920
NM_006416.3	*SLC35A1	N	0.1092139
NM_001032289.1	*SLC35A2	N	0.4035703
NM_005660.1	*SLC35A2	N	0.2186857
NM_012243.1	SLC35A3	N	0.0539033
NM_017945.2	SLC35A5	N	0.0529277
NM_005827.1	SLC35B1	N	0.2833235
NM_178148.1	SLC35B2	N	0.2264324
NM_015948.2	SLC35B3	N	0.1767236
NM_032826.3	SLC35B4	N	0.1178945
NM_018389.3	SLC35C1	N	0.2895977
NM_024881.3	SLC35E1	N	0.0648925
NM_018656.2	SLC35E3	N	0.2897321
NM_001001479.2	SLC35E4	N	0.2493643
NM_025181.2	SLC35F5	N	0.7687470
NM_078483.2	SLC36A1	N	0.9496541
NM_152313.2	SLC36A4	N	0.0834017
NM_207113.1	*SLC37A3	N	0.2869258
NM_138570.2	*SLC38A10	N	0.2277148
NM_018976.3	SLC38A2	N	0.5425033
NM_153811.1	SLC38A6	N	0.1327976
NM_018231.1	SLC38A7	N	0.3134832
NM_173514.1	SLC38A9	N	0.6683261
NM_139177.2	SLC39A11	N	0.7887703
NM_144564.4	*SLC39A3	N	0.2954138
NM_213568.1	*SLC39A3	N	0.4621386
NM_001077516.1	SLC39A7	N	0.3628948
NM_018375.2	SLC39A9	N	0.7813367
NM_001012661.1	SLC3A2	N	0.8618213
NM_001013251.1	SLC3A2	N	0.9633075
NM_001013251.1	SLC3A2	N	0.1123675
NM_173854.4	SLC41A1	N	0.2935131
NM_152346.1	SLC43A2	N	0.5313866
NM_080546.3	*SLC44A1	N	0.5037186
NM_032794.1	SLC44A4	N	0.3700385
NM_032034.2	SLC4A11	N	0.2116575
NM_018158.1	SLC4A1AP	N	0.9707707
NM_133478.2	SLC4A5	N	0.5828695
NM_003615.2	SLC4A7	N	0.3288379
NM_145913.2	SLC5A8	N	0.4965397
NM_014037.2	SLC6A16	N	0.7617007
NM_001076785.1	SLC7A6	N	0.2114797
NM_001076785.1	SLC7A6	N	0.4173361

NM_032178.1	SLC7A6OS	N	0.0679866
NM_003047.2	SLC9A1	N	0.0613602
NM_001042537.1	SLC9A6	N	0.3067372
NM_015266.1	SLC9A8	N	0.2146750
NM_007256.2	SLCO2B1	N	0.2149093
**ATP binding cassette transporters**			
**ACCESSION**	**SYMBOL**	**FOLD-CHANGE OR REGULATION**	**ADJ.P.VAL**
NM_005502.2	ABCA1	-1.410	0.000919475
NM_001089.1	ABCA3	-1.395	0.000936566
NM_000927.3	ABCB1	-1.249	0.0085752
NM_012089.1	ABCB10	-1.316	0.001893257
NM_019624.2	*ABCB9	-1.853	0.000236479
NM_005688.2	ABCC5	-1.352	0.001139969
NM_001023587.1	ABCC5	-1.323	0.000992726
NM_000350.2	ABCA4	1.138	0.046183334
NM_004299.3	ABCB7	1.273	0.001864064
NM_001040876.1	*ABCE1	1.225	0.016003879
NM_001025091.1	ABCF1	1.329	0.002154351
NM_001025091.1	ABCF1	1.735	1.04E-05
NM_007188.2	ABCB8	+	0.014799813
NM_000033.2	ABCD1	+	7.68E-06
NM_172232.1	ABCA5	N	0.47307086
NM_080284.2	ABCA6	N	0.744457735
NM_080284.2	ABCA6	N	0.315224209
NM_019112.3	ABCA7	N	0.114068852
NM_005689.1	ABCB6	N	0.598213725
NM_019624.2	*ABCB9	N	0.051314322
NM_019900.2	ABCC1	N	0.149614312
NM_033450.2	ABCC10	N	0.361807389
NM_003786.2	ABCC3	N	0.089941544
NM_005845.2	ABCC4	N	0.950285958
NM_001040876.1	*ABCE1	N	0.091359441
NM_005692.3	ABCF2	N	0.310193982
NM_007189.1	ABCF2	N	0.370861094
NM_018358.2	ABCF3	N	0.176371222
NM_016818.2	ABCG1	N	0.086265054
NM_016818.2	ABCG1	N	0.333291472

- Indicates downregulation below detection levels in cytokine-treated cells, + indicates upregulation from below detection levels in control cells, N indicates no changes between control and cytokine-treated cells and * indicates transcript showing different results with different probes.
